# Changed inflammatory markers after application of 4DryField PH for adhesion prevention in gynecological surgery

**DOI:** 10.1007/s00404-021-06095-7

**Published:** 2021-08-06

**Authors:** Nicole Ziegler, Luz Angela Torres-de la Roche, Rajesh Devassy, Rudy Leon De Wilde

**Affiliations:** Pius Hospital, University Hospital for Gynecology, University Medicine Oldenburg, Carl von Ossietzky University, Georgstrasse 12, 26121 Oldenburg, Germany

**Keywords:** Peritoneal adhesion, Starch-based polysaccharide, Adhesiolysis, Inflammation markers, Adhesion prophylaxis, Hemostasis

## Abstract

**Introduction:**

The development of peritoneal adhesions and the effects of different antiadhesion agents on such mechanisms are not fully understood. Temporary rises of the C-reactive protein (CRP) level have been reported after antiadhesion agent application. We present the changes of inflammation markers observed after use of a starch-based polysaccharide certified for adhesion prevention and hemostasis 4DF (4DryField^®^ PH).

**Method:**

Retrospective comparative analysis of inflammation markers in 40 patients undergoing laparoscopic adhesiolysis with or without adhesion prophylaxis was conducted. Statistical comparisons were performed by means of paired or unpaired *t* tests (for normally distributed continuous data), Wilcoxon matched pairs signed-rank tests or Mann–Whitney tests (for not-normally distributed continuous data), Mantel–Cox tests (for continuous data describing time intervals), and Fisher’s exact tests (for discrete data).

**Results:**

The maximum post-operative CRP level was significantly elevated in the 4DF group (87 vs. 29%; *p* < 0.001), whereas leukocyte concentration and body temperature did not differ between groups. No signs of infection were detected in any of the patients and CRP levels spontaneously dropped to normal values within few days. No side effects or complications were observed in both groups. In second-look surgeries performed for other diagnoses 1–56 weeks after the first interventions, no remnants of 4DF or any peritoneal inflammatory reactions were observed.

**Conclusion:**

The starch-based polysaccharide 4DF can be considered safe and does not induce inflammatory reactions of clinical significance. Further studies regarding 4DF degradation are recommended and, apart from macrophage migration, could also examine corresponding markers such as IL-6 and PCT.

## Introduction

When peritoneum is exposed to surgical trauma, multiple pro-inflammatory mechanisms are activated that can lead to peritoneal adhesions and fibrosis. The development of adhesions and the effects of different antiadhesion agents on such mechanisms are not fully understood [1].

4DryField^®^ PH (4DF) is a medical device, certified for adhesion prevention and hemostasis. It comes as a powder and is based on highly purified potato starch that has been modified. As a powder, 4DF provides hemostasis. The powder is hydrophilic and upon contact absorbs the watery components from blood thereby concentrating all components for the coagulation cascade, as well as the cells that partake in clot formation and, therefore, accelerates hemostasis. For adhesion prevention, the powder has to be mixed with saline solution. The product then forms a gel, which acts as a temporary barrier separating wounded surfaces, thereby preventing formation of adhesions between them. After about 7 days, the product is completely resorbed by the body. Previous publications documented the hemostyptic and adhesion prophylactic potency [2–4].

The instructions for use (IFU) of 4DF state that potentially a temporary rise of the C-reactive protein (CRP) level can occur after its usage. This observation is reviewed and quantified in the present retrospective, controlled study. Diagnostically, CRP is mainly used as an—unspecific—inflammatory marker. To differentiate if possibly occurring increased CRP values indeed are an isolated event in response to the medical device or if the patients showed an inflammatory reaction after all, further relevant parameters of inflammation, elevated body temperature and leukocytosis, were analyzed in this study.

The normal CRP level seen in most healthy adults is less than 0.3 mg/dl. Levels between 0.3 mg/dl and 1.0 mg/dl mean that a minor elevation is present, but this can be seen in obesity, pregnancy, depression, diabetes, common cold, gingivitis, periodontitis, sedentary lifestyle, cigarette smoking, and genetic polymorphisms. Levels above 1.0 mg/dl on the other hand can be seen as a sign of systematic inflammation, and from 10 mg/dl upwards, they can be a sign of an acute bacterial or viral infection [5].

Leukocytosis is generally considered to be present when the white cell count exceeds 11 nl^ −1^ [6] as several studies determining leukocyte ranges in healthy subjects found that this represents the 97.5‰ in these [7].

A systemic inflammatory response after surgery is (among others) defined by a body temperature above 38.0 °C [8]. Elevated body temperature alone is no sign of inflammation or infection though as it may occur after surgery even in their absence as the trauma elicits the production of pyrogenic cytokines.

## Materials and methods

A retrospective analysis of inflammation markers (CRP, leukocyte count, and body temperature) was conducted for 40 patients who underwent laparoscopic adhesiolysis with or without adhesion prophylaxis was conducted between November 2012 and August 2016 at Pius Hospital Oldenburg, Germany. The present analysis included patients who underwent only adhesiolysis as well patients who underwent adhesiolysis and additional procedures pertaining to the small pelvis and/or abdominal cavity. Information about the presence of adhesion at second surgeries performed for any reason up to 18 months of first surgery was also analyzed. To ensure equal groups, additional data were collected and compared between the groups.

Statistical comparisons were carried out employing paired or unpaired *t* tests (for normally distributed continuous data), Wilcoxon matched pairs signed-rank tests or Mann–Whitney tests (for not-normally distributed continuous data), Mantel–Cox tests (for continuous data describing time intervals), and Fisher’s exact tests (for discrete data). The level of significance (*α*) always was 0.05.

This investigation was part of another study, approved by the Ethics Committee of the European Medical School Oldenburg–Groningen (No. 129/2016) and has been performed in accordance with the ethical standards of the 1964 Declaration of Helsinki and its later amendments. The described blood values and parameters are part of the standard in perioperative care in the surgeries performed.

## Results

From 40 patients, 23 patients received no adhesion barrier and 17 were treated with 4DF gel. Patients of the 4DF group received an amount of 3 or 5 g of the product (depending on the wound size to be covered), applied as a powder and subsequently dripped with saline solution or premixed as a gel. All surgically affected surfaces in the peritoneal cavity were covered with the adhesion barrier.

Some of the patients had additional procedures performed, including resection of deep infiltrating endometriosis (48% of the patients in the control group, 41% in the 4DryField group), myomectomy (26%, 35%), total extirpation of ovaries (9%, 0%), adnectomy (9%, 0%), one sided adnexa removal (4%, 6%), as well as singular patients (4%) in the control group with ovarian cystectomy, ovarian tumor, tumourectomy (right adnexa), abscess drainage or salpingectomy, and singular patients (6%) in the 4DF group with laparoscopic assisted vaginally tumor resection (intramural uterine tumor, resection of ovarian fibroids), deperitonealization of the small pelvis, suture of sigmoid colon, or tumorectomy of the pelvic wall. In addition, 27 patients of control group and 20 patients of 4DF group underwent a second-look surgery within 18 months of first procedure.

The mean surgery duration was 74 ± 43 min in the control and 104 ± 67 min in the 4DF group, the mean age was 35.6 ± 7.6 years in the control and 42.9 ± 14.5 years, and the mean BMI was 24.7 ± 5.6 kg/m^2^ and 24.1 ± 3.3 kg/m^2^. Statistical comparison of these data, as well as the surgical procedures that were carried out in addition to adhesiolysis yielded no significant differences. Adhesions were scored during the intervention concerning their extent and severity using a 5-point scale from 0 to 2, including half-integral scores. The median values for the control group were 1 (extent) and 1 (severity), and for the 4DryField group, they were 2 (extent) and 2 (severity). Both these differences were statistically significant (*p* < 0.001).

The average post-operative maximum values for CRP, leukocyte count, and body temperature, as well as the values above the respective upper reference limits are given in Table 1. The single observation of a leukocyte level above 11.0 nl^ −1^ (which was 16.3 nl ^−1^) and the single occurrence of a body temperature above 38.0 °C (which was 38.2 °C) in the 4DF group refer to two different patients. No patient in the 4DF group had an increased leukocyte level plus an elevated body temperature. The elevated leukocyte counts in the control group were 14.5 and 16.6 nl^ −1^.

**Table 1:** Evaluation of the maximum post-operative C-reactive protein, leukocyte, and body temperature values

	Control group (*n* = 23)		4DryField group (*n* = 17)		*p*
	AM	SD	AM	SD	
CRP (mg/dl)	0.8	1.2	7.6	9.4	< 0.001*
Leukocytes (nl^ −1^)	4.9	4.6	6.4	4.2	0.431
BT (°C)	37.1	0.3	37.0	0.5	0.595

	*n* = 23	%	*n* = 17	%	
CRP > 1.0 mg/dl	6	29	13	87	< 0.001*
Leukocytes > 11.0 nl^ −1^	2	10	1	7	> 0.999
BT > 38.0 °C	0	0	1	7	0.4054

*AM* arithmetic mean, *SD*   standard deviation, *BT*   body temperature

*Statistically significant (*p* < 0.05)

The maximum post-operative CRP level was significantly elevated in the 4DF group, whereas leukocyte concentration and body temperature did not differ between both groups. No signs of infection of the surgical areas (i.e., redness, swelling, and exudate/purulence) were detected in any of the patients after laparoscopy and CRP levels spontaneously dropped to normal values within a few days and without any required treatment. No side effects or complications could be observed in both groups.

In second-look surgeries performed for other diagnoses 1–56 weeks after the first interventions, no remnants of 4DF or any inflammatory reactions were observed.

## Discussion

Available evidence shows a high level of adhesiogenesis associated with routine abdominal procedures. Protection against postsurgical adhesion formation lies in the prevention of acute inflammation in the peritoneal cavity and delays in its natural reparation process [1]. Therefore, antiadhesion barriers should have impact on factors derived from mesothelial cells and acute inflammation in the entire peritoneal cavity [1, 9].

For other adhesion prevention devices commonly used in gynecologic surgery, increased CRP values without leukocytosis or fever have not been prominently reported. However, there are reports of inflammatory responses towards other adhesion barriers. A barrier based on oxidized regenerated cellulose (Interceed) was shown to elicit a peritoneal fluid inflammatory exudate characterized by large number of activated macrophages [10] and histopathological analyses showed local inflammatory responses where remnants of the membrane persisted [11]. Similarly, peritoneal inflammation has been observed at the site of placement, as well as corresponding foreign body granuloma reactions have been seen for a barrier based on a mixture of hyaluronate and carboxymethylcellulose (Seprafilm) [12, 13]. For the same barrier, there is a report of inflammatory reaction caused by extensive adhesion formation, again in conjunction with a foreign body reaction [14]. 4DF is the only adhesion barrier that is based on modified starch, as well as the only one that comes in powder form, suggesting that one or both of these factors are possibly causative for this isolated CRP increase.

A previous study on 23 patients who underwent extensive surgery, either hysterectomy and/or resection of deep infiltrating endometriosis leaving large areas of peritoneal defects, reported no local infections and a low frequency of transitory high CRP levels after 4DF application. Here, 7% of cases had a CRP level up to 10 mg/dl (normal value < 0.5 mg/dl), which was accompanied by mild leukocytosis in two (13.1 nl^ −1^, 16.1 nl^ −1^) and elevated temperature (39.1 °C) in one patient [4].

As this study did not include a control group, it is hardly possible to estimate to which part these CRP increases were to be expected and which might be ascribable to 4DF usage.

In our cohort, a higher frequency of low-increased CRP values (> 1.0 mg/dl) was observed in the 4DF group (87 vs. 29%), which spontaneously dropped to normal values within few days. However, no increased leukocyte level plus an elevated body temperature was reported in the 4DF group.

Comparison of basic data suggests that comparability of the groups is given, as there were no statistically significant differences except for severity and extent of lysed adhesions. These were higher in the 4DF group, and thus, the CRP increase could possibly be ascribed in part to this difference. Nevertheless, the main result, i.e., higher CRP values in the 4DF group do not indicate inflammation, remains unaffected as this could only have been distorted if the values would have been higher in the control group.

Comparability between our and earlier results is complicated as the two patient collectives had different surgical interventions, so that differing CRP increases were to be expected. Additionally, reference values of a control group, which would make comparability easier, are missing for the other data set.

Our explanation for the rise of CRP levels values after application of 4DF for adhesion prevention could be the increased local concentration of macrophages in the area of 4DF application, as part of its degradation process [4, 15]. Macrophages are known to play an important role in the early phase of foreign body reactions, during which monocytes differentiate into classically activated macrophages and secrete pro-inflammatory cytokines, such as interleukin-6 (IL-6) [16, 17]. These interleukins, in turn, lead to an increased CRP level by stimulating the CRP synthesis, as well as an increased differentiation of monocytes to macrophages [16]. Correspondingly, immunological research over the past 2 decades has shown that neither IL-6 nor CRP are unambiguous inflammatory markers. Instead, it is supposed that both molecules participate in somatic maintenance efforts, with elevated levels indicating that an organism is investing in preservation, protection, and/or repair of somatic tissue [18].

In diagnostics, CRP is mainly used as an inflammatory marker. Its possible distortion after 4DF usage impairs its usability as an inflammatory marker. As the surgeon can still rely on leukocyte levels and body temperature development, as well as general signs, such as unusual pain, misdiagnoses should not be of concern, but the surgeon should be aware of the possible effect on CRP levels. Although the ephemerality of the effect on the CRP level, combined with the lack of additional signs of inflammation or infection, already enables a secure differentiation of the effect on the CRP level from a real inflammation or infection, the use of different laboratory markers for inflammatory reactions, such as procalcitonin (PCT), should be evaluated in further research. It is likely that such markers are not influenced in a similar way as CRP levels, since the mechanisms for the induction of their respective expression are different.

## Conclusion

Despite a transitory enhancement of the CRP values after application of 4DF for adhesion prevention in gynecological surgery, our results show that this starch-based polysaccharide can be considered safe and does not induce inflammatory reactions of further clinical significance. Further studies regarding 4DF degradation are recommended and, apart from macrophage migration, could also examine corresponding markers such as IL-6 and PCT.
